# Comparative Metabolomic Profiling Reveals Key Secondary Metabolites Associated with High Quality and Nutritional Value in Broad Bean (*Vicia faba* L.)

**DOI:** 10.3390/molecules27248995

**Published:** 2022-12-16

**Authors:** Shou-Heng Shi, Seung-Seop Lee, Ya-Ming Zhu, Zhu-Qun Jin, Fei-Bo Wu, Cheng-Wei Qiu

**Affiliations:** 1 Institute of Crop Science, College of Agriculture and Biotechnology, Zhejiang University, Hangzhou 310058, China; 2 Cixi Agricultural Technology Extension Center, Cixi 315300, China

**Keywords:** legume, germplasm resource, widely targeted metabolomics, LS-MS/MS, flavonoids, healthy eating

## Abstract

High quality and nutritional benefits are ultimately the desirable features that influence the commercial value and market share of broad bean (*Vicia faba* L.). Different cultivars vary greatly in taste, flavor, and nutrition. However, the molecular basis of these traits remains largely unknown. Here, the grain metabolites of the superior Chinese landrace Cixidabaican (CX) were detected by a widely targeted metabolomics approach and compared with the main cultivar Lingxiyicun (LX) from Japan. The analyses of global metabolic variations revealed a total of 149 differentially abundant metabolites (DAMs) were identified between these two genotypes. Among them, 84 and 65 were up- and down-regulated in CX compared with LX. Most of the DAMs were closely related to healthy eating substances known for their antioxidant and anti-cancer properties, and some others were involved in the taste formation. The KEGG-based classification further revealed that these DAMs were significantly enriched in 21 metabolic pathways, particularly in flavone and flavonol biosynthesis. The differences in key secondary metabolites, including flavonoids, terpenoids, amino acid derivates, and alkaloids, may lead to more nutritional value in a healthy diet and better adaptability for the seed germination of CX. The present results provide important insights into the taste/quality-forming mechanisms and contributes to the conservation and utilization of germplasm resources for breeding broad bean with superior eating quality.

## 1. Introduction

Broad beans (*Vicia faba* L.), one of the extensively cultivated legumes worldwide, are commonly grown as vegetables, ruminant feeds, cover crops, and green manures [1]. Its seeds have a high content of proteins, starch, and dietary fiber with beneficial components, such as lysine, L-Dopa, vitamins, minerals, and phenolic compounds, making this crop a rich source of vegetable protein and pharmaceutical products in some countries [2,3,4,5]. China has the largest broad bean production around the world, with an annual seed yield of about 1.8 Mt, accounting for 44% of the total [6]. The great market share of local broad bean has been occupied by the large-seeded cultivar Lingxiyicun (LX) from Japan since 1995, posing a serious threat to genetic diversity and breeding. As the secondary origin center of broad bean, China also possesses abundant resources of broad bean varieties and has more than 4000 years of cultivation history [7]. Cixidabaican (CX), an elite landrace in Zhejiang, used to be the most popular variety throughout China due to its high quality and excellent nutritional profile. In addition, CX also exhibits a wide range of adaptability in various unfavorable environmental conditions, such as disease, drought, and low temperature [8]. However, the molecular basis underlying these traits remains largely unknown.

Secondary metabolites are organic compounds derived from primary metabolism that modulate appearance, flavor, and taste, as well as stress resistance in plants [9,10]. Legumes are rich in secondary metabolites, such as polyphenols, alkaloids, and saponins [11,12]. Among them, polyphenols are the major determinants of tissue colors, while alkaloids and saponins are known for contributing to the bitter taste of plants [13,14]. In addition, polyphenols have multiple functions in plants for the alleviation of oxidative damage, contributing to adaptive traits and ecological fitness [15]. There are also a wide range of secondary metabolites with nutraceutical and pharmaceutical values for humans. Widely distributed in most legume seeds, flavonoids and anthocyanins are important antioxidant and anti-inflammatory compounds for human health, serving key roles in reducing cardiovascular disease and body weight [16,17]. Besides polyphenols, the high level of carotenoids also acts as an antioxidant that provides protection for the eye from damage by free radicals [18]. Catechin has shown to be beneficial in reducing heart disease and improving sperm motility and viability [19,20]. Nevertheless, the loss of diversity and secondary metabolites in modern cultivars limits the potential of legumes as sources of bioactive compounds and also renders the plants more susceptible to abiotic and biotic stress.

Over the last few years, metabolomics approaches have emerged as powerful and reliable tools for the rapid detection of a wide range of plant metabolites, complementary to transcriptomic and proteomic analyses in functional genomics and systems biology [21]. A recent widely targeted metabolomics analysis of soybean and chickpea revealed different advantages between the two legumes and new functional compounds for diabetes, proving this an effective way for dissecting the genetic and biochemical basis of plant metabolism [22]. The comparative untargeted metabolome profiling established the fingerprinting between legumes, including chickpea, lentil, and white bean, which provides new biomarkers to fight against food fraud [23]. Another comparative metabolomics study of drought acclimation in model and forage legumes uncovered conserved and unique metabolic responses to drought stress [24]. In addition, several studies on the metabolome profiling of legume crops identified key metabolites, such as amino acids, alcohols, and alkaloids, were involved in signaling pathways under insect stress and fungal pathogens [25,26]. So far, there is very little information available on the metabolites of common legumes, and a complete phytochemical profile of broad bean is still elusive.

Our previous agronomic and proteomic analyses demonstrated CX had higher pod numbers and yield per plant than LX and identified many differentially abundant proteins related to carbohydrate and amino acid metabolism for storage duration and stress resistance in CX [8]. Thus, we hypothesized that the superior traits of CX are associated with its metabolic composition. Here, widely targeted metabolomics analyses were used to investigate the global difference of metabolites between CX and LX. The objective of the present study was to identify key metabolites associated with high quality and nutritional value, understand the substances of these excellent traits in CX, and extend our knowledge on the improvement in broad bean breeding and conservation programs.

## 2. Results

### 2.1. Widely Targeted Metabolite Profiling of CX and LX

**Figure 1:**
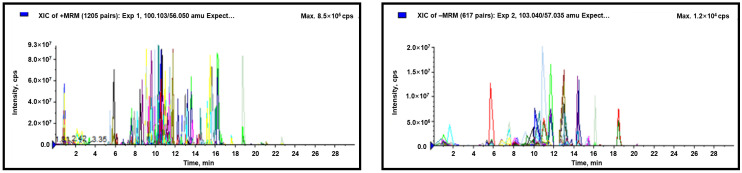
Multi-peak plot of metabolites in MRM (multiple reaction monitoring) detection. Each color represents a different metabolite.

To explore the comprehensive variations in the metabolomes of two contrasting broad bean genotypes, a widely targeted analysis was applied by high-throughput liquid chromatography–tandem mass spectrometry (LC-MS/MS) approaches, and the relative contents of metabolites were analyzed by the MRM (multiple reaction monitoring) mode using MultiQuant version 3.0.2 (AB SCIEX, Concord, ON, Canada) software (Figure 1).

**Figure 2:**
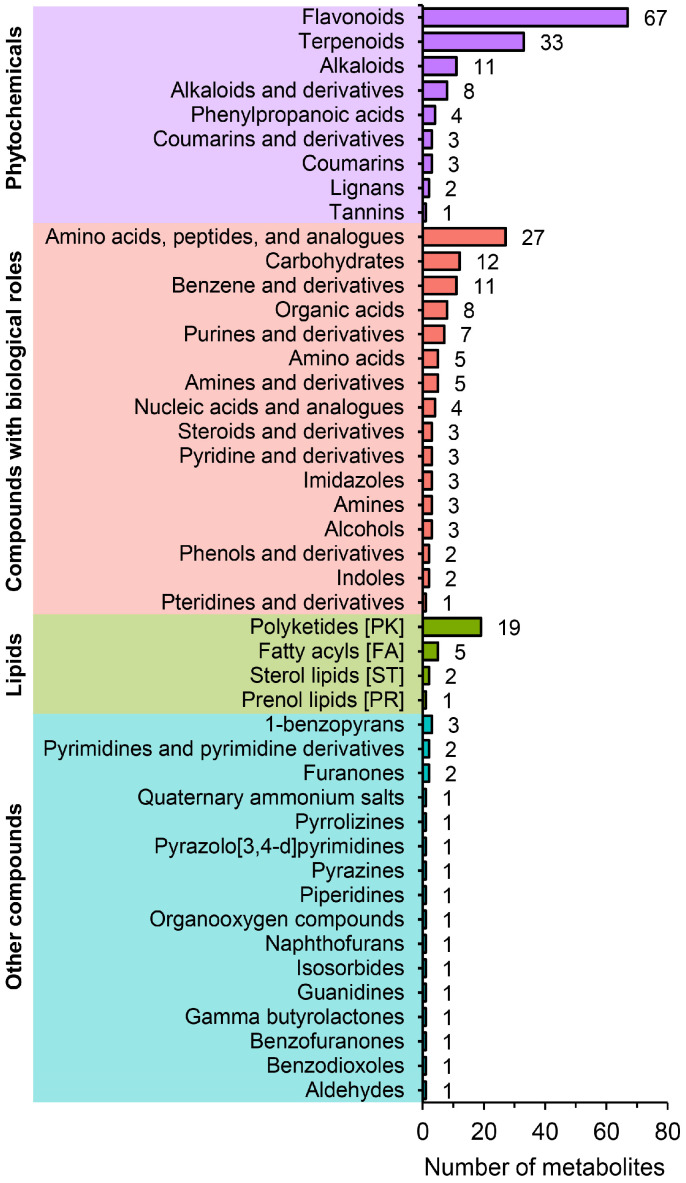
Identification and classification of the metabolites from CX and LX.

A total of 434 metabolites were identified in CX and LX. Among them, 278 known metabolites can be divided into 45 categories, including 32 amino acids and analogues, 8 organic acids, 27 lipids, 4 nucleotides, 12 carbohydrates, 175 secondary metabolites (composing flavonoids, alcohols, alkaloids, etc.), and 20 other metabolites (Figure 2). The number of flavonoids in secondary metabolites was the highest. In addition, some lipids that are considered beneficial to human health such as jujuboside B and crocin II were detected.

**Figure 3:**
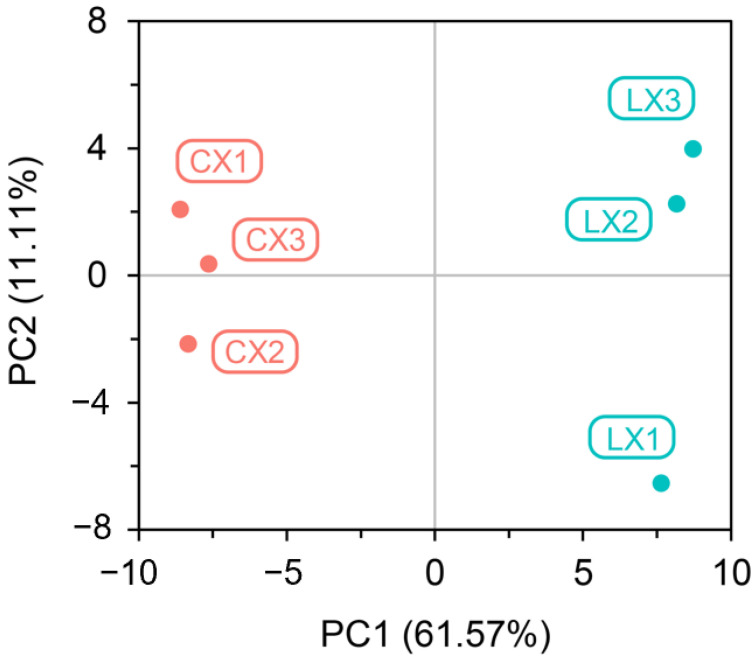
Principal component analysis (PCA) of the metabolites from CX and LX.

Pareto scaling and principal component analysis (PCA) were performed on samples to provide an initial understanding of the overall metabolic differences between the groups of samples and the degree of variability between samples within the group. Additionally, the percentages of explained values in the metabolome analysis of PC1 and PC2 were 61.57% and 11.11%, respectively, indicating PC1 resolved the separation of CX and LX (Figure 3). According to the PCA score, the metabolites of the two genotypes can be divided into three obviously separated sample groups, which indicates that there were large differences in metabolite composition. In addition, the samples in one group were aggregated together with less variation. 

### 2.2. Identification and Clustering of Differentially Abundant Metabolites

**Figure 4:**
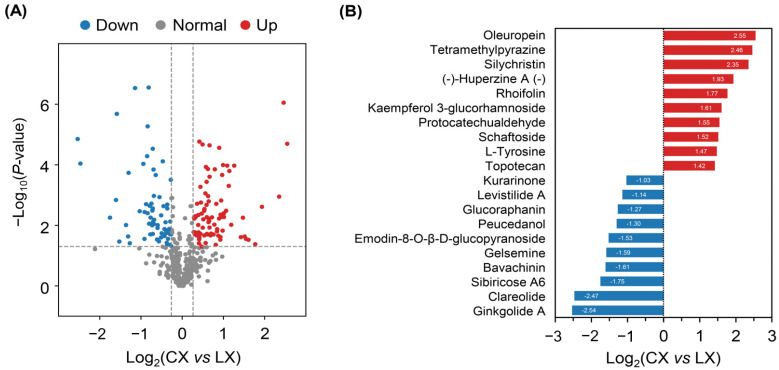
Identification of differentially abundant metabolites (DAMs). (**A**) Volcano plots of DAMs between CX and LX. (**B**) Top 20 most significant DAMs.

The metabolite abundances of all samples were quantified by log2 ratio and normalized by zero-mean. Comparative analyses with all annotated metabolites were subsequently conducted by applying the supervised PLS-DA model to identify the significant metabolites between CX and LX. The differentially abundant metabolites (DAMs) between the two genotypes were determined based on variable importance in projection (VIP) ≥1 and the fold-change ≥1.2 or ≤0.83 with *p*-value < 0.05. A total of 149 DAMs were detected, accounting for 53.60% of the total metabolites, indicating great differences in metabolites between CX and LX (Figure 4A; Table S1). Among them, 56.38% of DAMs were up-regulated in CX, whereas the rest were down-regulated, accounting for 43.62%. In addition, 20 metabolites were important in distinguishing CX and LX, in which oleuropein, tetramethylpyrazine, silychristin, (-)-huperzine A (-)-, rhoifolin, kaempferol-7-O-β-D-glucopyranoside, protocatechualdehyde, schaftoside, L-Tyrosine, and topotecan were predominantly accumulated in CX, and ginkgolide A, clareolide, sibiricose A6, bavachinin, gelsemine, emodin-8-O-β-D-glucopyranoside, peucedanol, glucoraphanin, levistilide A, and kurarinone were predominantly accumulated in LX, respectively (Figure 4B). 

**Figure 5:**
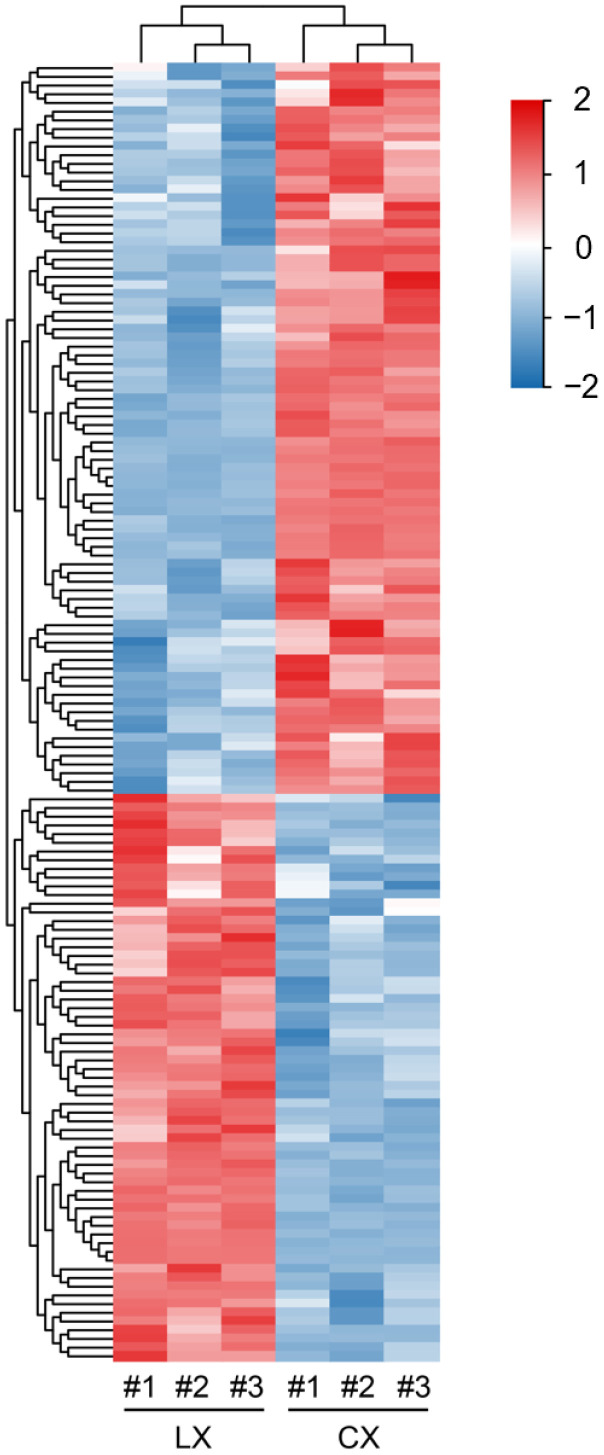
The hierarchical clustering analysis of DAMs between CX and LX. The heatmap of DAMs was displayed by Euclidean distance and complete cluster methods as a measurement of similarity.

Furthermore, all DAMs were grouped by hierarchical clustering and Pearson correlation analysis. The CX predominantly accumulated metabolites were grouped into Cluster I, and the LX predominantly accumulated metabolites were grouped into Cluster II, with remarkable variations consistently with PCA data (Figure 5).

### 2.3. Annotation and Functional Classification of DAMs

**Figure 6:**
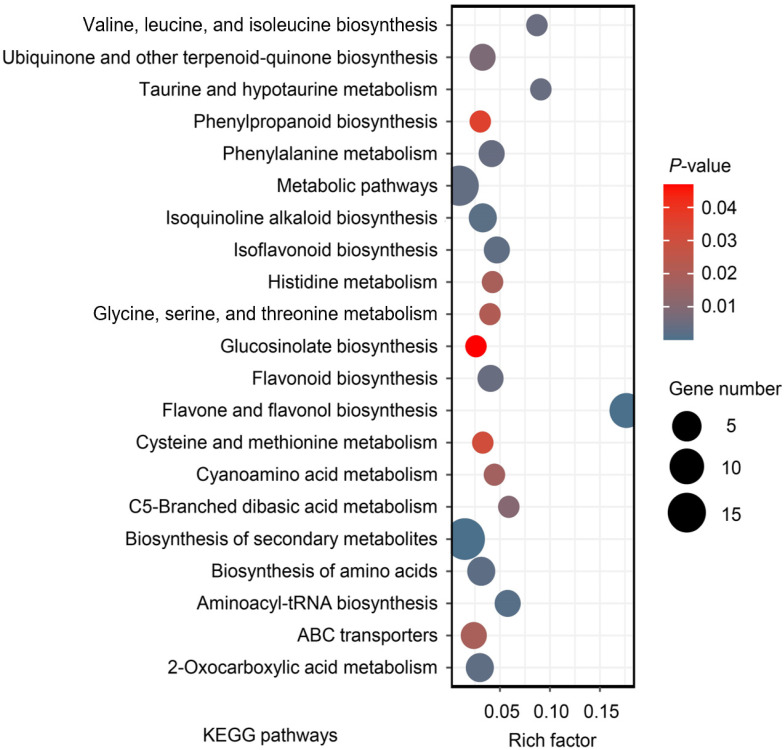
Enrichment analysis of DAMs between CX and LX based on Kyoto Encyclopedia of Genes and Genomes (KEGG).

To provide a deep overview of the metabolic variations, all DAMs were annotated and functionally classified according to Kyoto Encyclopedia of Genes and Genomes (KEGG). A total of 121 metabolites were assigned to 48 metabolic pathways, and the major enrichment of KEGG pathways included flavone and flavonol biosynthesis, the biosynthesis of secondary metabolites, aminoacyl-tRNA biosynthesis, isoquinoline alkaloid biosynthesis, the biosynthesis of amino acids, and isoflavonoid biosynthesis (Figure 6). Flavone and flavonol biosynthesis were the most significant pathways between CX and LX. In these pathways, nine metabolites were detected by widely targeted metabolomics. Among them, seven metabolites (rutin, rhoifolin, lonicerin, astragalin, quercitrin, apigenin-7-O-β-D-glucoside, and isoquercitrin) were significantly accumulated in CX, while isovitexin and cynaroside were significantly accumulated in LX.

### 2.4. Key Secondary Metabolites Associated with High Quality and Nutritional Value

**Figure 7:**
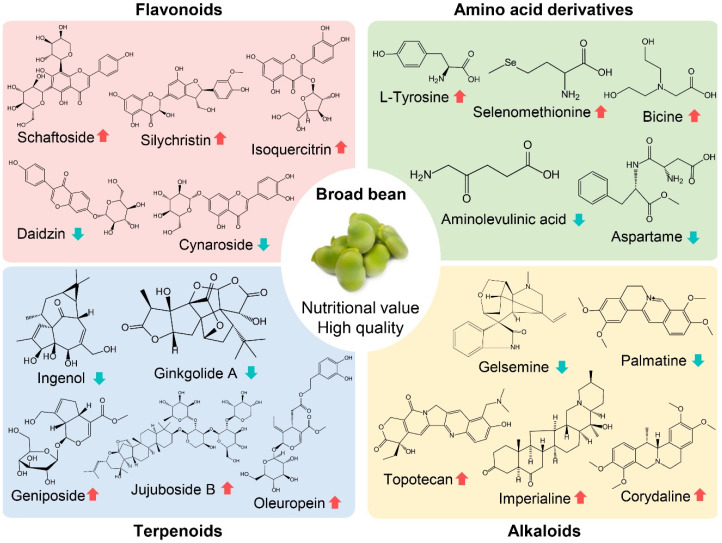
Key secondary metabolites related to quality, nutrition and stress resistance in broad bean. Arrows indicate up- (red) and down-regulation (cyan) of the corresponding metabolite in CX, respectively.

Many secondary metabolites have been shown to be related to quality, nutrition, and stress resistance in legumes, so we further determined the distinction of these metabolites between the two varieties, including 28 flavonoids, 13 terpenoids, 11 amino acid derivates, and 5 alkaloids (Figure 7; Table S2). Most flavonoids, such as silychristin and isoquercitrin, were up-regulated in CX, whereas others, such as daidzin and cynaroside, were down-regulated. Many terpenoids were far more abundant in CX than in LX, including oleuropein, geniposide, and jujuboside. There was a considerable difference in the relative content of several amino acid derivates (L-tyrosine, L-(+)-selenomethionine, aspartame, and 5-aminolevulinic acid). In addition, among alkaloids, topotecan, imperialine, and corydaline were up-regulated in CX, while gelsemine and palmatine were down-regulated. Moreover, one of three amines (5-methoxytryptamine), two of four benzenes (4-hydroxybenzoic acid and protocatechualdehyde), one organic acid (guanidinoethyl sulfonate) and two carbohydrates (D-erythronic acid and D(-)-salicin) were also up-regulated in CX.

## 3. Discussion

Broad bean is one of the main legumes grown worldwide and plays crucial roles in human diet and health. Significant progress has been made to understand the physiological and molecular basis in broad bean breeding for resistance to drought, salinity, disease, and pest [27,28,29]. Recently, there has been growing interest in the development of broad bean enriched with bioactive compounds with improved quality, nutritional value, and health benefits [30]. For example, efforts have been made on the removal of antinutritive compounds in broad bean seeds, and the low content of tannins and vicine have been recognized as an indicator of food safety and better taste [31]. The different cultivars of broad bean plants can contain variable metabolites. However, not much information is currently available about a comprehensive wide-scale metabolomic perspective of broad bean seeds, particularly in Chinese landraces. Here, the primary and secondary metabolites of CX and LX were identified and quantified by widely targeted metabolomics analysis. A total of 434 metabolites were obtained from these two varieties. Of these, 89 metabolites were significantly enriched in CX, whereas 65 metabolites were prominently decreased compared to LX, mainly including flavonoids, terpenoids, amino acids, and lipids, indicating these substances may be closely relevant to the outstanding quality of CX (Figure 4 and Figure 5). Our dataset provides the foundation to understand the molecular basis of the important quality and nutrient traits in commercial legumes.

In this study, the composition and contents of the major kinds of secondary metabolites flavonoids varied greatly between the two broad bean varieties (Figure 6; Table S1). Flavonoids comprise a large and diverse group of polyphenolic compounds and are an integral part of the human diet, with antioxidant, hypoglycemic, hypolipidemic, and anticancer properties [32]. There is increasing evidence that dietary flavonoids are likely candidates for the observed beneficial effects of a diet rich in fruits and vegetables on the prevention of several chronic diseases, reducing the risk of cancer in the digestive tract [33], skin, and sexual organs [34,35]. We found that flavonoids with anticancer and anti-inflammatory effects were highly accumulated in CX, including rutin [36], astragalin [37], quercitrin [38], isoquercitrin [39], rhoifolin [40], and lonicerin [41], which confers the high quality and nutritional benefits of this elite landrace. In plants, flavonoids are widespread throughout all stages of growth and development, playing important roles in many biological processes, such as the pigmentation of flowers, fruits, and vegetables, plant–pathogen interactions, fertility, and protection against abiotic stress [42]. As non-enzymatic antioxidants, flavonoids are also involved in seed germination for signaling and ROS scavenging [43]. We found many DAMs enriched in flavone and flavonol biosynthesis pathways (Figure 5), which may indicate that the seeds of CX are able to survive under varying environmental conditions compared to LX and have better performance to cope with drought, heavy metals, salt, radiation, and other stresses. 

Besides metabolites in the flavone and flavonol biosynthesis pathways, our widely targeted metabolome analyses uncovered more functional bioactive compounds in CX than LX (Tables S1 and S2; Figure 6). The content of oleuropein, tetramethylpyrazine, and silychristin in CX were five times more than those in LX. Among them, oleuropein is another kind of important phenolic compound, which is also known for its pharmacological potential with a number of beneficial effects, such as antioxidants, anti-cancer, antiviral, and anti-aging [44,45,46]. Although with health benefits, the bitter nature of oleuropein may affect the taste and flavor of CX [47]. Similarly, several metabolites that were up-regulated about 2.5 times in CX are also involved in nutritional benefits and pharmacological application, including rhoifolin [48], protocatechualdehyde [49], topotecan [50], kaempferol 3-glucorhamnoside [40], and schaftoside [51]. In addition, huperzine A, a significantly up-regulated metabolite in CX, is an inhibitor of acetylcholinesterase and is a benefit to the nervous system, which has been used in many countries for the treatment of Alzheimer’s disease [52,53]. However, we found that the content of the flavoring metabolite clareolide was lower in CX, which may also explain the different tastes between CX and LX [8]. 

## 4. Materials and Methods

### 4.1. Plant Materials

Two broad bean (*Vicia faba* L.) varieties, Cixidabaican (CX, a local landrace commonly grown in Cixi, Zhejiang Province, China) and Lingxiyicun (LX, introduced from Japan in 2017), were grown under natural field conditions at the experimental station of Cixi Agricultural Technology Extension Center (30.02° N, 121.02° E), Ningbo, China. Single-seed dibble sowing was used, with 1.0 m row spacing and 0.35 m plant spacing. Nine replications were established with 16 plants per plot (2.8 m × 2 m), each containing two rows. The mature seeds of CX and LX were harvested with three replicates, and each replicate sample contained three independent biological replicate samples, which were then placed in liquid N_2_ immediately, then stored at −80 °C until further analysis.

### 4.2. Sample Preparation and Extraction for Metabolomics Analysis

One-hundred-milligrams of freeze-dried seed powder was weighted and was mixed with 1.0 mL of precooled 70% aqueous methanol and triturated in a grinder (50 Hz, 5 min), followed by an overnight extraction at 4 °C. The supernatants from the centrifugation (13,000 r min^−1^, 4 °C, 10 min) were absorbed and filtrated (SCAA-104, 0.22 μm pore size; ANPEL, Shanghai, China) for LC-MS/MS analysis.

### 4.3. High-Performance Liquid Chromatography Conditions

For each genotype, three biological replicates were independently analyzed. The sample extracts were analyzed using a LC-MS/MS system. A UPLC system (Waters, Milford, MA, USA) equipped with a QTRAP 6500 Plus mass spectrometer (SCIEX, Redwood City, CA, USA) was used for the separation and quantitative analysis of the samples; the analytical conditions were as follows: the mobile phase consisted of solvent A (0.1% formic acid aqueous solution) and solvent B (0.1% formic acid acetonitrile). Elution was carried out at a flow rate of 0.3 mL/min with the following gradient: 0~2 min, 5% solvent B; 2~22 min, 5~95% solvent B; 22~27 min, 95% solvent B; 27.1~30 min, 95% solvent B; and the column temperature was thermostatically controlled at 40 °C. QTRAP 6500 Plus mass spectrometer was equipped with an Est Turbo Ion Spray interface; the source operation parameters were set as follows: source temperature, 500 °C; ion spray voltage (IS), 5500 V (positive)*/*−5500 V (negative); ion source gas I (GSI), gas II(GSII), and curtain gas (CUR) were set at 40, 40, and 25 psi, respectively; and the MRM method in MRM mode was adopted, which included the metabolite information, collision energy (CE), de-clustering voltage (DP), and retention time of the target metabolite.

The quality control (QC) samples were prepared from all the sample extracts to become a combined sample, then divided and analyzed using the same method as the experimental samples. The QC samples were injected every ten experimental samples throughout the analytical run to provide a set of data from which repeatability could be assessed.

### 4.4. Qualitative and Quantitative Analysis of Metabolites

The identification and quantitative analyses were based on the BGI-wide Target-Library; extracted ion chromatograph (XIC) was obtained. Mass spectra and metabolite data analysis were conducted with Analyst 1.6.3 software (AB Sciex, Framingham, MA, USA), whereby retention time, mass–nucleus ratio, and peak intensity were available, and each peak of a particular color represented a metabolite. All of the peak areas were integrated, and the same metabolite in different samples were corrected through MultiQuant version 3.0.2 (AB SCIEX, Concord, ON, Canada), which was used to determine the relative metabolite contents. PCA (principal component analysis), PLS-DA (partial least squares method–discriminant analysis), and univariate analysis, such as fold-change (FC) and Student’s *t*-test, were used to evaluate the model. Differentially abundant metabolites (DAMs) were identified by the fold-change (≥1.2 or ≤0.8333), *p*-value (<0.05) from the univariate analysis, and variable importance in the projection (VIP ≥ 1) values of the first two principal components in the PLS-DA model. All DAMs were annotated and functionally classified according to Kyoto Encyclopedia of Genes and Genomes (KEGG) and then mapped to the KEGG Pathway database; pathways with *p*-value < 0.05 were determined as pathways with a significant enrichment of DAMs.

## 5. Conclusions

In this study, the widely targeted metabolic profiling of the Chinese landrace CX and Japanese-introduced cultivar LX with a difference in grain quality was investigated through LC-MS/MS analysis. The PCA and clustering of the metabolic dataset showed clear distinctions between these two genotypes. Furthermore, the PLS-DA model unveiled a total of 149 DAMs that may work cooperatively to establish a complex metabolic network for the taste formation and nutrient composition of broad beans. More importantly, the metabolites with beneficial effects on human health, such as oleuropein, silychristin, tetramethylpyrazine, rhoifolin, protocatechualdehyde, and topotecan, were identified as the key metabolites related to high quality and nutritional value in broad bean; most of them have higher enrichment in CX, which suggests that CX may have better nutritional qualities than LX. This study demonstrated the excellent characteristics of CX, providing important insights of native elite germplasm resources for future conservation and utilization.

## Data Availability

The data presented in this study are available in article and Supplementary Materials.

## Supplementary Materials

The following supporting information can be downloaded at: https://www.mdpi.com/article/10.3390/molecules27248995/s1, Table S1: Identification and characterization of DAMs between CX and LX; Table S2: Key DAMs associated with quality, nutrition, and stress resistance in broad bean.
